# Computational–Experimental Design Framework for Laser Path Length Controller

**DOI:** 10.3390/s21155209

**Published:** 2021-07-31

**Authors:** Tevfik Ozan Fenercioğlu, Tuncay Yalçinkaya

**Affiliations:** Department of Aerospace Engineering, Middle East Technical University, Ankara 06800, Turkey; ozan.fenercioglu@metu.edu.tr

**Keywords:** path length controller, piezoelectric actuator, laser interferometry, low force gauge, laser triangulation, finite element analysis, mode-scanning

## Abstract

The application areas of piezoelectric materials are expanding rapidly in the form of piezo harvesters, sensors and actuators. A path length controller is a high-precision piezoelectric actuator used in laser oscillators, especially in ring laser gyroscopes. A path length controller alters the position of a mirror nanometrically by means of a control voltage to stabilize the route that a laser beam travels in an integral multiple of laser wavelength. The design and verification of a path length controller performance requires long (up to 3 months), expensive and precise production steps to be successfully terminated. In this study, a combined computational–experimental design framework was developed to control, optimize and verify the performance of the path length controller, without the need for ring laser gyroscope assembly. A novel framework was structured such that the piezoelectric performance characteristics were calculated using finite element analysis. Then, a stand-alone measurement system was developed to verify the finite element analysis results before system integration. The final performance of the novel framework was verified by a direct measurement method called mode-scanning, which is founded on laser interferometry. The study is concluded with the explanation of measurement errors and finite element correlations.

## 1. Introduction

Nowadays, piezoelectric materials are widely used in industrial applications. The main principle relies on the piezoelectric effect, which is the induction of an electrical charge under mechanical strain and vice versa. Piezoelectric materials have found emerging application areas in energy harvesting and actuation. Energy harvesting is a method developed to generate energy from vibrations on piezo elements. The most significant applications include implantable and wearable power supplies, energy harvesting from ambient fluid flows, microelectromechanical systems and self-powered sensors. These applications are reviewed in detail in the literature (see [1,2,3]). Recent studies focus rather on self-powered systems and on low-energy electronics. The correlation between the force applied and the energy induced and its linear attitude showed a significant potential for measurement systems. Piezoelectric sensors are developed for acceleration and vibration measurements after the reveal of piezoelectric effects (see [4,5,6]).

Similarly, another application has emerged from piezoelectric properties: it was observed that the material straining under the application of an electrical field makes it possible to use as an actuator. By changing shape, stack orientations and polarization, different piezo actuators have been developed such as piezoelectric bending elements (benders), piezo ceramic tubes, rings, etc. Piezoelectric bending elements are a popular branch of piezoelectric actuators because of the amplification in piezoelectric forces. In order to construct a piezoelectric bender, two piezoelectric materials are bonded on the upper and lower surfaces of a beam. In this way, one side elongates while the other side shortens to create a bending motion. This hybrid structure is also applied for different geometries. Some examples of piezoelectric actuators for precise applications are as follows: microactuators (see [7]), ultrasonic motors (see [8]), active vibration control (see [9]), path length controller (PLC) of laser systems (see [10,11]), dither mechanism of ring laser gyroscopes (RLGs) (see [12]), low force sample holders (see [13,14]), accelerometers and constant frequency vibration sources.

**Figure 1:**
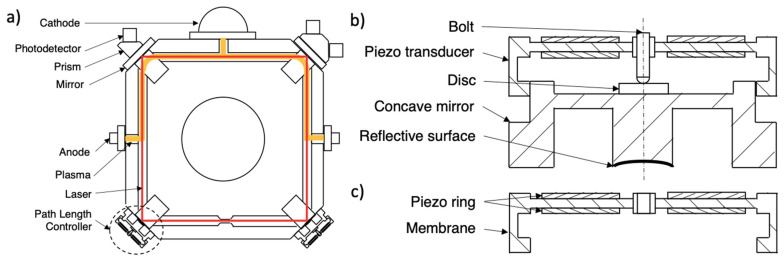
Schematics of (**a**) RLG structure, (**b**) PLC structure and (**a**,**c**) piezo transducer. Adapted from ref. [17].

Since piezoelectric actuators are used in precise positioning applications, their design processes require detailed calculation, analysis and tests. In this study, a novel design framework was structured through finite element analysis and tests of the piezoelectric response for piezo transducers (precise piezo actuators) of path length controllers within a laser oscillator, namely the ring laser gyroscope (RLG). Before explaining the novel design framework, information about the system requirements of laser gyroscopes is briefly given in the proceeding section. Figure 1a shows the schematic structure of a 4-mirror RLG. For most of the RLGs, laser beams are emitted inside a cavity by enforced plasma reactions (when an external laser source is used, the sensor is named as a solid-state RLG). Low pressure He-Ne gases are used inside the cavity. When an electrical discharge is applied between anodes and cathodes, this energy changes the state of the cavity gases. Moreover, continuous state changes inside the cavity results in spontaneous emission, which is the main laser source [15]. However, emissions caused by state variations cannot result in full functional laser waves. In order to amplify laser light and indirectly sensor output signals, a resonance condition on laser wavelength is required. 

Inside the RLG cavity, the laser beam travels a predetermined route. As the laser wave propagates inside the cavity, it interacts with reflective surfaces to reflect and complete the closed shape. The total distance between orthogonal reflective surfaces is defined as the laser path length. The resonance condition is obtained when the laser path length is an integral multiple of laser wavelength. When this condition is satisfied, laser amplitude tends to amplify and the amplified signal can be used for a wide range of applications such as distance measurement, rotation measurement by RLG, etc. Otherwise, when this condition is not satisfied, the laser wave will interfere with itself which suppresses the laser power and stability [16]. Numerically, the wavelength of the He-Ne laser is ~632.8 nm. Holding path length as an integral multiple of the 632.8 nm is a demanding problem since even the smallest temperature variation results in a deflection of the path length because of the thermal expansions. Hence, a path length controller is used in order to compensate for path length variations. It is a precise position control system where a concave mirror is attached to a piezo transducer (assembly of the piezoelectric ring actuator). Some additional motion transfer elements (bolt and disc) are used to transfer the displacement created on the piezo transducer to the concave mirror (shown on Figure 1b). The piezo transducer controls the position of the mirror’s center nanometrically by means of a control voltage. It stabilizes the path length as an integral multiple of laser wavelength.

A piezo transducer is an actuator with two piezoelectric rings that are attached on upper and lower surfaces of a metal ring similarly to a piezoelectric bender. The outer surface of the metal ring is fixed. Hence, the center of the ring makes vertical motion when an electrical field is applied to the piezoelectric rings. This motion is transferred to the mirror to control the position of the reflective surface (see Figure 1). 

A deep optimization is not possible using analytical expressions and individual test results of piezoelectric rings because the PLC assembly has adhesively bonded parts, which alters the total stiffness and limits kinematics of the structure. Moreover, the predetermination of the performance characteristics of a piezoelectric actuator is an essential requirement for the system design.

In the literature, different design and optimization studies are conducted for PLC design. These studies mostly consider the role of stiffness on deflections. Designs of the membrane and mirror are optimized to maximize vertical deflections and to minimize horizontal deflections, also called mirror-tilt (see [17,18,19]). 

One of the most important shortcomings in these approaches is that analysis studies are not conducted including piezoelectric effects. Verifications of these design studies are made after the RLG is assembled using the mode-scanning test. This requires long (up to 3 months), expensive and precise production steps to be successfully terminated.

**Figure 2:**
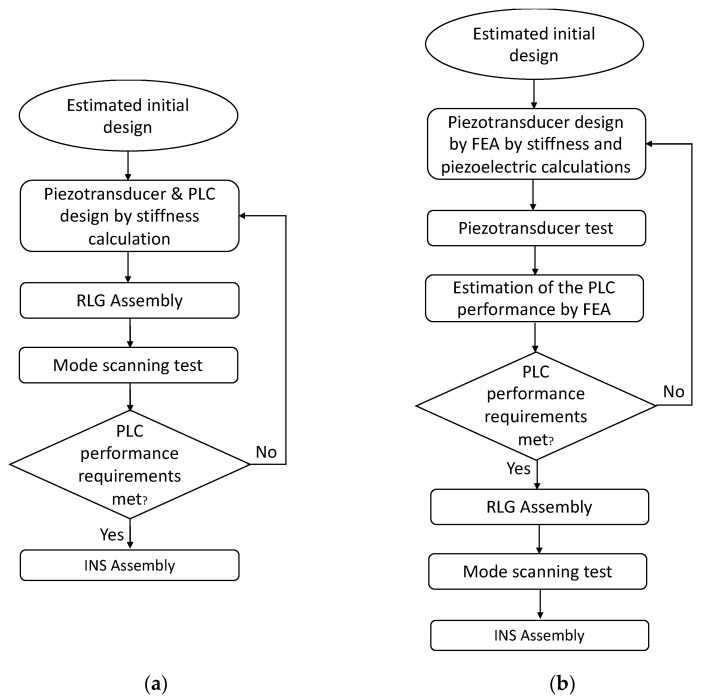
Flowcharts of the PLC design framework: (**a**) conventional; and (**b**) combined computational–experimental frameworks.

In this study, a combined computational–experimental design framework was developed to solely control, optimize and verify vertical deflections of the PLC without the need for RLG assembly. Both conventional and novel computational–experimental design approaches are illustrated in Figure 2. A novel framework (shown in Figure 2b) was structured such that the performance characteristics of a piezo transducer within a path length controller were calculated individually by suppressing peripherals using finite element analysis (FEA). Then, a stand-alone measurement system was developed to verify FEA results before system integration. After the evaluation of the subsystem results, a second finite element model was developed for the path length controller of the laser oscillator. In this way, it was validated by FEA that the path length controller design provides the RLG system requirements. The mode-scanning test after RLG assembly was only used to calibrate INS electronics. However, during the conventional production method (shown in Figure 2a), there was no information or method to estimate PLC performance. Therefore, PLC performance validation can only be made after RLG assembly with a mode-scanning test. The resulting system is a unique contribution to the literature as it validates the numerical methods and enforces current validation techniques within design processes.

After presenting the introduction here, Section 2 addresses the design of the piezo transducer and its finite element analysis. Section 3 studies the piezo transducer test bench. Using the results of computations and experiments, PLC performance is estimated in Section 4. The estimated performance is validated using direct measurement in Section 5. Then, the study is concluded in Section 6 with the explanation of measurement errors and finite element correlations, which is believed to be valuable for the future design and optimization studies of precise piezoelectric applications.

## 2. Piezo Transducer Design

In this section, the novel design framework is pursued for a sample piezo transducer geometry. Initially, numerical simulations were performed to reveal the kinematics of the piezo transducer. Finite element analysis (FEA) was preferred for simulations; it is a powerful approximation technique to predict the real-time response of the materials and structures, where the different governing differential equations are solved in domains divided into small regions in integral forms.

The first model was constructed to examine piezo transducer response individually with the mirror and disc suppressed. Hence, the technique was used to calculate vertical displacements of the bolt tip of the piezo transducer. The nonlinear FEA solver MSC.Marc^®^ was used in this study. Axisymmetric analysis was used and 4-node quadrilateral elements were employed. Piezoelectric-compatible finite element formulation was selected. Real piezoelectric rings contained a thin layer of conductive coating. This layer was coated in order to uniformly distribute the applied electrical potential. In FEA, electrical potential is uniformly distributed to all related nodes. Hence, the conductive layer is neglected.

**Figure 3:**
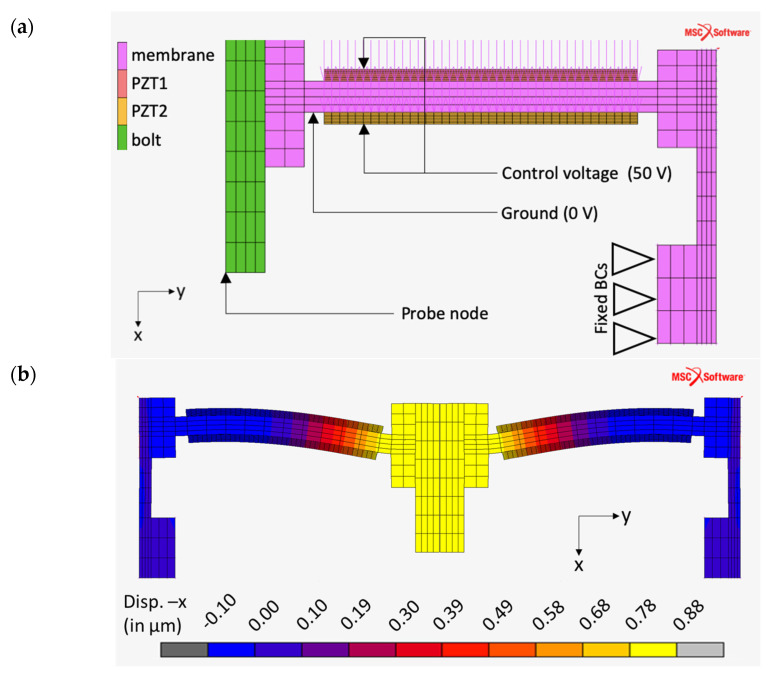
Finite element analysis of the piezo transducer. (**a**) Model definition and (**b**) vertical displacement results.

The piezo transducer model and analysis results are visualized in Figure 3. For the piezo transducer analysis model, the displacements and rotations on all directions were restricted for the membrane lower arm. Finite element analysis of the PLC structure showed no significant displacement on this region, which makes it a favorable area for fixed boundary conditions. Thus, this surface was made the contact surface for the piezo transducer test fixture. A fixed electrical potential of 50 V on the free surface of the piezoelectric ring and 0V fixed electrical potential (ground) on the mating surfaces between the piezoelectric rings and metal membrane were applied for both models. Finally, piezoelectric materials were introduced using a combined mechanical and electrical field constitutive law (stress-based). Details of the combined constitutive law are given below (see [20,21] for more details).

The constitutive equations of the mechanical response and the piezoelectric responses are presented below:

(1) σ=L∶ε−e·E

(2) $$D=e^{T}∶\varepsilon +\xi^{\varepsilon }\cdot E$$

Here,

σ is the stress tensor, unit: N/m^2^.

ε is the strain tensor, unit: m/m.

L is the elastic stiffness, unit: N/m^2^.

D is the electric displacement vector, unit: C/m^2^.

E is the electric field vector, unit: V/m.

e is the piezoelectric matrix (stress-based), unit: N/Vm.

ξ is the permittivity, unit: F/m.

Due to testing capabilities, material properties are strain-based, defined by manufacturers. The strain-based form of constitutive equations and electrostatic response are given below:

(3) ε=C∶σ+d·E

(4) $$D=d^{T}∶\sigma +\xi^{*}\cdot E$$

Here,

C is the elastic compliance, unit: m^2^/N.

d is the piezoelectric matrix (strain-based), unit: m/V.

ξ* is the permittivity (strain-based), unit: F/m

The strain-based properties are converted to stress-based properties by MSC.Marc^®^ through following relations:

(5) e=L∶d

(6) $$\xi =\xi^{*}-e^{T}\cdot d$$

Another important aspect of the piezoelectric analysis is the coordinate definition. For piezo rings of the PLC, a cylindrical coordinate system is used where thickness direction is defined as the polarization axis.

Finally, a two-index notation is used by manufacturers to identify piezoelectric properties. The first index means the direction of the electrical field and the second is the mechanical behavior written in the Voigt notation presented below:

(7) $${\left[\begin{array}{c} \sigma_{1}\\ \sigma_{2}\\ \sigma_{3}\\ \sigma_{4}\\ \sigma_{5}\\ \sigma_{6} \end{array}\right]}^{voigt}=\left[\begin{array}{c} \begin{array}{c} \sigma_{11}\\ \sigma_{22}\\ \sigma_{33}\\ \sigma_{23}\\ \sigma_{13}\\ \sigma_{12} \end{array} \end{array}\right]$$

(8) $${\left[\begin{array}{c} \varepsilon_{1}\\ \varepsilon_{2}\\ \varepsilon_{3}\\ \varepsilon_{4}\\ \varepsilon_{5}\\ \varepsilon_{6} \end{array}\right]}^{voigt}=\left[\begin{array}{c} \begin{array}{c} \varepsilon_{11}\\ \varepsilon_{22}\\ \varepsilon_{33}\\ 2\varepsilon_{23}\\ 2\varepsilon_{13}\\ 2\varepsilon_{12} \end{array} \end{array}\right]$$

Using the relevant coordinate system, strain-based constitutive equations can be written in the matrix form [21] as below:

(9) $$\left[\begin{array}{c} \varepsilon_{1}\\ \varepsilon_{2}\\ \varepsilon_{3}\\ \varepsilon_{4}\\ \varepsilon_{5}\\ \varepsilon_{6} \end{array}\right]=\left[\begin{array}{cccccc} C_{11} & C_{12} & C_{13} & 0 & 0 & 0\\ C_{12} & C_{22} & C_{23} & 0 & 0 & 0\\ C_{13} & C_{23} & C_{33} & 0 & 0 & 0\\ 0 & 0 & 0 & C_{44} & 0 & 0\\ 0 & 0 & 0 & 0 & C_{55} & 0\\ 0 & 0 & 0 & 0 & 0 & C_{66} \end{array}\right]\left[\begin{array}{c} \sigma_{1}\\ \sigma_{2}\\ \sigma_{3}\\ \sigma_{4}\\ \sigma_{5}\\ \sigma_{6} \end{array}\right]+\left[\begin{array}{ccc} 0 & 0 & d_{31}\\ 0 & 0 & d_{32}\\ 0 & 0 & d_{33}\\ 0 & d_{24} & 0\\ d_{15} & 0 & 0\\ 0 & 0 & 0 \end{array}\right]\left[\begin{array}{c} E_{1}\\ E_{2}\\ E_{3} \end{array}\right]$$

(10) $$\left[\begin{array}{c} D_{1}\\ D_{2}\\ D_{3} \end{array}\right]=\left[\begin{array}{cccccc} 0 & 0 & 0 & 0 & d_{15} & 0\\ 0 & 0 & 0 & d_{24} & 0 & 0\\ d_{31} & d_{32} & d_{33} & 0 & 0 & 0 \end{array}\right]\left[\begin{array}{c} \sigma_{1}\\ \sigma_{2}\\ \sigma_{3}\\ \sigma_{4}\\ \sigma_{5}\\ \sigma_{6} \end{array}\right]+\left[\begin{array}{ccc} \xi_{11}^{*} & 0 & 0\\ 0 & \xi_{22}^{*} & 0\\ 0 & 0 & \xi_{33}^{*} \end{array}\right]\left[\begin{array}{c} E_{1}\\ E_{2}\\ E_{3} \end{array}\right]$$

Table 1 presents anisotropic piezoelectric properties defined in the analyses. These properties were taken from [22] for C-601 type PZT (Pb(ZrTi)O3) material. The elastic properties of other parts are presented in Table 2.

When piezoelectric rings are loaded with 50 V, 0.863 µm displacement is obtained on the central node of the bolt tip of the piezo transducer. The 500× magnified views of the deformation fields of the piezo transducer is shown in Figure 3b. This result was exported after a mesh convergence study was conducted. It was observed that the element lengths of the piezo rings especially have significant influence. A cross-sectional view of the ring was obtained by mirroring over y-plane symmetry. It should be noted that the deformation mode of piezoelectric rings is similar to piezoelectric benders.

For most of the RLG designs, a control range of 3 integral multiples of wavelength (3 × 632.8 nm = 1898.4 nm = 1.8984 µm) over 200 V (control voltage) is required from PLCs to ensure RLG system performance. The results of the initial design (0.863 µm/50 V) may look like an overdesign but it should be noted that the additional stiffness of mirrors can reduce this range dramatically. However, this result of the initial FEA study was effectively used in this framework to define requirements of the piezo transducer test bench (e.g., measuring range).

## 3. Piezo Transducer Test

**Figure 4:**
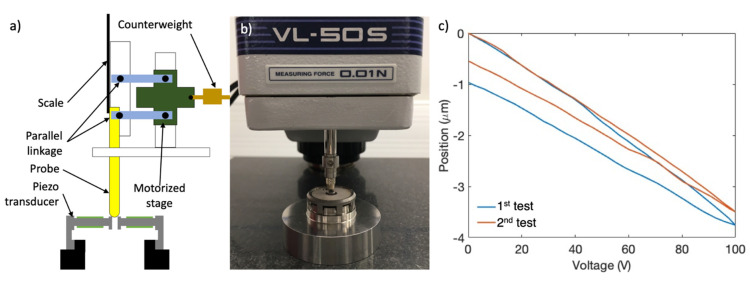
Schematics and results of the contact-based measuring technique. (**a**) Schematics of the parallel linkage system used in Litematic VL-50S-B; (**b**) image of the piezo transducer test bench; and (**c**) piezo control voltage–probe position figure.

Using the results of the FEA, at least 1 µm/50 V measuring capacity with 0.01 µm resolution was requested from the test system. Initially, a conventional contact-based measuring technique was applied for practical reasons. This method finds applications in static thickness measurement of wafers and electronics. Mitutoyo Litematic VL-50S-B (Japan) was used in order to measure the vertical deflection of the piezo transducer, which is a low-force (0.01 N), high-resolution (0.01 µm) motorized vertical displacement measuring tool. In this system, the probe moves down via a motorized stage. As the probe touches the measuring surface, it moves parallel linkage up to a stop position and the motor stops [23]. To measure the motion of the piezo transducer, the measuring probe initially touched on the upper surface of the sub-assembly and the scale was set to zero at this position. The deviations of the measuring probe when the piezo elements were loaded by control voltage are given in Figure 4.

After 2 consecutive tests, −1.75 and −1.66 µm displacements were obtained under 50 V using contact-based measuring. A reference control voltage of 50 V was made for this study because reference RLGs usually change one laser mode under approximately 50 V. Displacement values were higher than expected. Moreover, a significant hysteresis was observed on the loading–unloading curves. The responses did not coincide and a shift up to 0.96 µm was obtained. There might be various reasons for the discrepancy. First, there was a contact force (0.01 N) between the probe and piezo transducer. Although, this force is quite small, it might be relatively dominant over piezo forces. Furthermore, this system was used for static measurements in general. Therefore, a drift of the measuring probe causing instability on the results was considered to have influence on the results. 

Additionally, further evaluation of the finite element analysis revealed several effects of the boundary conditions. The restriction of the displacements and rotations using fixed boundary conditions was not reflected in the stand-alone test system due to the lack of adhesive bonding. In the initial test system, the metal legs of the piezo transducers were adhesively bonded to the mirror (glue contact was applied between parts of the PLC FEA model). This was also applied in the piezo transducer analysis model where displacements and rotations on all directions were restricted for the metal legs. Therefore, vertical motion on the outer sections was supported for both conditions (PLC and piezo transducer). However, in reality, these DOFs were free to shift vertically on the stand-alone test system.

After this observation, voltage–position curves were considered to be invalid. Then, a further literature study was conducted for a better stand-alone test system. In the new test system, a better correlation for the kinematics of the PLC was aimed between the stand-alone test system and final product assembly. The investigation has revealed that the laser displacement meters are practical solutions for position control of piezo structures [24]. The triangulation principle was employed in these sensors instead of interferometry. A reference laser was reflected over the measured surface, then the reflections were focused by a lens and collected on a charge-coupled device (CCD, photodetector). The CCD output signal was calibrated relative to the position of the reflections. Hence, displacement of the measured surface changed the position of the reflections on the CCD element. In this way, using the CCD output, displacement was measured.

**Figure 5:**
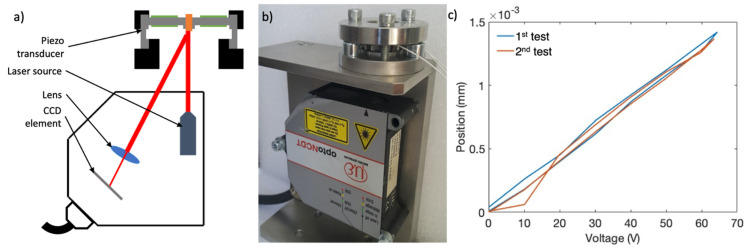
Schematics and results of the laser measuring technique. (**a**) Schematics of the laser triangulation; (**b**) image of piezo transducer test bench; and (**c**) piezo control voltage–CCD position figure.

A non-contact laser measurement system was assembled for the piezo transducer, which is illustrated in Figure 4. A dummy bolt was attached to the structure to create the reflective face needed (shown in orange in Figure 4a). A laser triangulation sensor (Type: OptoNCDT 2300, Micro-Epsilon GmbH, Germany) was used for this system with a resolution of 0.03 µm [24]. Additionally, an upper cap was manufactured and bolted on the upper surface of the piezo transducer to restrict the vertical shift of the legs (shown in black in Figure 5a). In this way, the free shift of the metal legs was restricted and missing boundary conditions were supplied.

The results for this device are shown in Figure 5. After 5 consecutive tests, approximately 1 µm displacement was measured for 50 V. Thus, approximately 15% error was calculated between the test and FEA results. The results of the non-contact displacement test were regarded to be more reliable since the loading–unloading curves closely coincide individually and similar slopes (2 × 10^−5^) were calculated after a linear fitting. Reliability of the measurement was provided by the improvements of the novel test system. First of all, by using a non-contact measurement technique, excess measurement load is removed. Then, by using FEA results, the effects of the boundary conditions and kinematics of the structure are revealed and a realistic fixture is designed.

The compatibility of the trend between the initial FEA and test results for the piezo transducer made it possible to estimate PLC performance with FEA. It was anticipated that a safety factor could be set by considering the margin of error in the final product design. Additional efforts to reduce the error were also carried out and will be discussed in detail in the conclusion section.

## 4. Estimation of the PLC Performance

**Figure 6:**
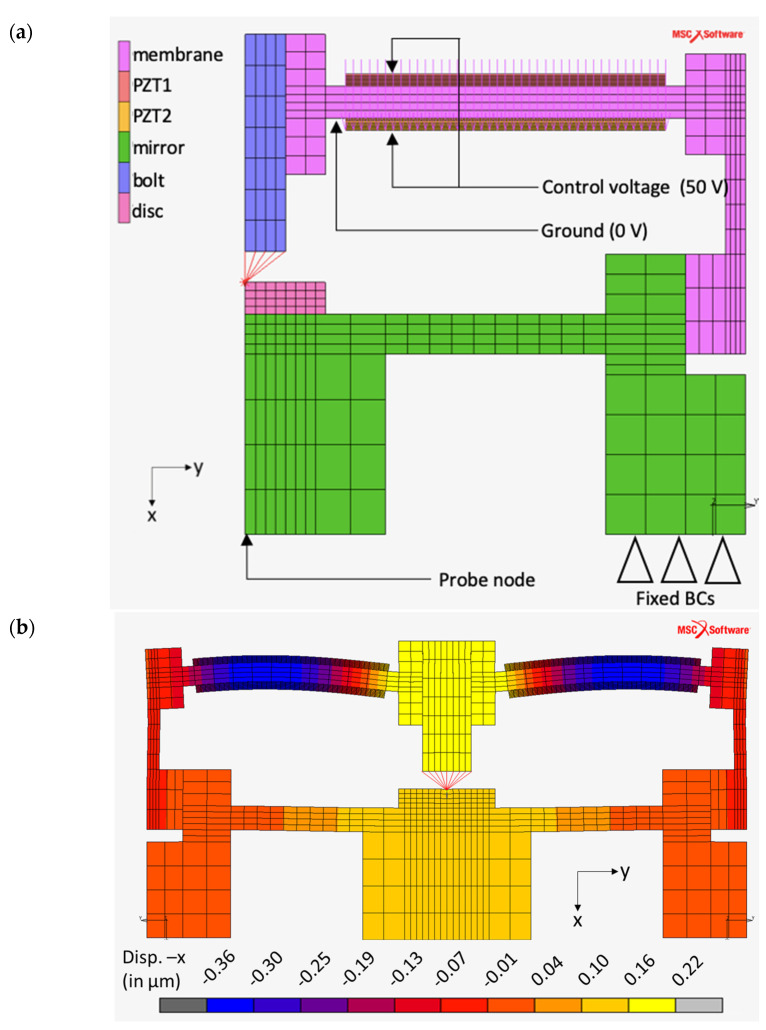
Finite element analysis for the PLC. (**a**) Model definition; (**b**) vertical displacement results.

In this section, numerical simulations are performed to reveal the kinematics of the PLC and effects of peripheral parts on the PLC performance. Finite element analysis (FEA) was preferred as explained in Section 2. The PLC model and analysis results are visualized in Figure 6a. In the PLC structure, the main parts include the piezo transducer and mirror. The mirror and piezo transducer were adhesively bonded. A glue contact (in MSC.Marc^®^) was selected to the model for adhesive bonding of the piezo transducer over the mirror. This type of contact colligates the degree of freedom for nodes on both parts’ surfaces. By using this method, even though the stiffness of the adhesive was overestimated, a significant divergence was not expected on the validity of the final results because the load level was relatively small during this section and the adhesive application was strictly regulated. Additionally, a bolt and disc (peripherals) were used to transfer the motion between the piezo transducer and mirror. Initially, the motion was transferred from the piezo transducer to the bolt. Then, the bolt would push or pull the disc. The disc was mainly used to distribute the force uniformly over the mirror. The modelling of these parts also requires special attention for model reliability. PLC bolt tips are designed spherically to have point contact on the disc. Additionally, the disc was adhesively bonded to the upper surface of the mirror. Instead of modelling spherical tip geometry, translation was transferred using small rigid beam elements (RBE2). Thus, the deflection of the tip (0.2 mm height) was neglected. A glue contact (in MSC.Marc^®^) was selected to model adhesive bonding of the disc over the mirror. Similar to the glue contact between the piezo transducer and mirror, a significant divergence was not expected on the validity of the final results because the load was small and mostly compressive during this section and the adhesive application was strictly regulated. For the PLC analysis model, the displacements and rotations on all directions were restricted for the lower mirror surface. Finally, piezoelectric materials were introduced using a combined mechanical and electrical field constitutive law (stress-based) as explained in Section 2.

Since the most important reflection region is the center of the PLC mirror, vertical displacement was exported from FEA for this region to estimate the performance. When piezoelectric rings were loaded with 50 V, 0.129 µm displacement was obtained for the PLC assembly. The 80× magnified views of the deformation fields of PLC are shown in Figure 6b.

Larger displacement which was obtained in the initial model is an expected result since mirror and other peripherals are suppressed, and the total vertical stiffness of the structure is reduced. By exporting the vertical deflection of the center node for the mirror, validation of the FEA was ensured since this vertical motion is testable through a direct test method, namely mode-scanning. It should be noted that vertical displacements determined by mode-scanning are a combined response of the piezo transducer, concave mirror and other peripheral parts.

## 5. Mode-Scanning Test

In this section, a reliable reference measurement technique is specified. The results of the reference measurement are used to evaluate the outputs of the previous sections. The reference measurement of the current study relies on laser interferometry. Interferometry is based on a laser source and its beam which is divided using a beam splitter. After splitting, the first beam is kept intact within a known distance, while the second reflects from the measuring surface. Afterwards, a second beam splitter combines the reflections of both signals. Interference patterns between the reference laser beam and measurement beam are tracked and correlated to real distance through a photodetector [18,19]. Examples in the literature use separate laser source units. Hence, due to assembly tolerances, a small amount of position error is introduced. However, the laser oscillator used in this study was a RLG which contains a built-in laser thus higher precision was expected (less tolerance accumulation).

In this study, a 4-mirror RLG was constructed to verify the performance of the PLC. Direct measurement was conducted by the mode-scanning test. As explained above, the amplitude of the laser signal varies with the path length and when the path length is an integral multiple of the wavelength, maximum signal is achieved.

**Figure 7:**
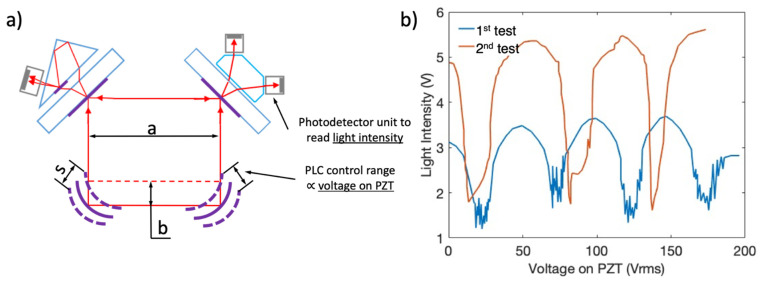
(**a**) Mode-scanning test schematic of a 4-mirror laser oscillator where the initial path is a square of pathlength, (4a) which changes to (4a–2b) after a linear perturbation, (s/2) of 2 PLCs; (**b**) figure shows the fluctuations of the light intensity as a function of PZT voltage.

In the literature, it has been stated that a √2 λ/4 vertical motion of a PLC mirror creates an integral λ (632.8 nm) arrangement on the laser path length [10]. This relation is used to determine the correlation between the laser amplitude and control voltage of the PLC. The laser amplitude is recorded by changing the control voltage of the PLC. Then, the voltage difference between two maxima of the laser amplitude is assumed to be an integral wavelength change of the path length (Figure 7). For an integral wavelength change, 0.112 µm vertical displacement on the mirror center is expected. The local maxima of the laser amplitude are called the laser mode and the process to determine the laser signal amplitude as light intensity by scanning the control voltage of the path length controller is called the mode-scanning test. Figure 7 shows the test configuration and the results. It was observed that ~48 V on the piezoelectric elements created an integral mode change for the current oscillator.

The results are numerically summarized in Table 3. The relation between positions of the local maxima for laser amplitude and control voltage of the PLC was examined and ~0.117 µm/50 V was probed by means of a mode-scanning test. Thus, approximately 10% error was calculated between the test and FEA results. Error sources and future efforts to reduce them are explained in the conclusion section.

## 6. Discussion

In this study, the novel computational–experimental framework structures a systematic evaluation method for PLC performance without conducting RLG assembly. It has been revealed that the determination of piezoelectric performance of the piezo transducer (sub system) by FEA and tests can be used to estimate the final performance of the PLC (main system) after the addition of an acceptable safety margin (10%). This safety margin was defined after the comparison of the PLC FEA results with the mode-scanning test. The mode-scanning test was especially preferred to improve the precision of the reference measurement state by means of a built-in laser instead of a separate laser source unit. Two main reasons were identified for the margin given: simulation-based and test-based disparities.

Simulation-based disparities are defined as the differences between real parameters and numerical modelling approaches. Although piezoelectric analysis is a mature analysis type, it is only reliable upon using accurate material properties, which are not available for commercial piezo elements of every grade and shape. The expected displacement was 0.129 µm/50 V from the FEA results. The mode-scanning test resulted in 0.117 µm/50 V displacement. After examining and improving the modeling parameters (mesh convergence check, etc.), the reason behind the difference between the analysis and test results was attributed to the material properties. The material properties used in the analysis are presented in bulk forms in the specification documents. However, the shape, grade, production method and polarization affected the final displacement response of the piezoelectric elements. Hence, this contributes to the 10% error since a commercial grade piezo ring was used. It has been stated in the literature that usually a calibration (rearrangement of d_31_ in equation 9 to decrease the error) is necessary by experiments to prevent variations of properties [25]. However, this calibration was not applied in this study for consistency between the two analysis models. The results after this type of calibration are only valid when the same material is used within the study. Otherwise, a recalibration is necessary when the material changes, which is impractical for optimization studies. Moreover, a small portion of the error is thought to arise from the use of an axial symmetric FEA model. Figure 4b shows that the sample piezo actuator has eight discrete legs. When an axial symmetric model is used, legs are assumed to be continuous. The 3D construction of the same model extends the solution times to an unacceptable level for a design framework due to the very small dimensions of the finite elements (piezoelectric-compatible) used for the piezo ring. Therefore, the use of an axial symmetric FEA model is recommended.

Test-based disparities resulted from differences between the end product and stand-alone test configuration. As explained in the preceding sections, a mode-scanning test is accepted as the most reliable method in order to characterize the real response of the structure because the position errors due to tolerance accumulations are minimized. However, since most of the parts are bonded (undetachable after testing) to create a hermetical assembly to construct a laser oscillator (RLG), this method requires all assembly operations to be performed before the test, which is not practical for serial production. The stand-alone test system was designed such that the PLC is detachable after each test. Despite this advantage, error sources were included in the system: mounting tolerances and their accumulation. The type of fit, used between the test bench and PLC, contributes to the difference between the mode-scanning and stand-alone test results. A transition fit with a clearance of up to 40 μm was selected between the test bench and PLC. This fit is recommended for optical parts requiring full concentricity. Also, a small portion of the error is thought to arise from the mechanical assembly of the stand-alone test system.

Moreover, the presented disparities do not contain large uncertainties. Hence, the safety margin can be accepted as a reference value for further design studies. Overall, it has been proven that the novel method works for PLC design evaluation and has managed to shorten the long (up to 3 months), expensive and precise production steps. 

## Figures and Tables

**Table 1:** Piezoelectric material properties.

Mat. Name	Piezoelectric Strain COEFFICIENT (×10^−12^ C/N)			Elastic Modulus (×10^10^ N/m^2^)			Relative Permittivity		Density (kg/m^3^)
	d31 = d32	d33	d15 = d24	C11	C33	C55	ξ_11_	ξ_33_	
C-601	−210	500	730	6.7	5	2.2	3130	3400	7500

**Table 2:** Material properties.

Part Name	Material	Density (kg/m^3^)	Young’s Modulus (Pa)	Poisson’s Ratio
Mirror	ULE Glass	2530	9.03 × 10^10^	0.24
Membrane, bolt	Invar Alloy	8100	1.4 × 10^11^	0.28
Disc	40CrNiAl	8100	2.2 × 10^11^	0.28

**Table 3:** Numerical results.

Part Name	Test Results [µm]			FEA Results (% Error) [µm]
	Contact Based	Laser Triangulation	Laser Mode-Scanning	
Piezo transducer	1.7 *	1	-	0.863 (15%)
PLC	-	-	0.117	0.129 (10%)

* considered to be invalid due to error rates and hysteresis.

## References

[1] Anton S.R., Sodano H.A. (2007). A review of power harvesting using piezoelectric materials (2003–2006). Smart Mater Struct..

[2] Kunz J., Fialka J., Pikula S., Benes P., Krejci J., Klusacek S., Havranek Z. (2021). A New Method to Perform Direct Efficiency Measurement and Power Flow Analysis in Vibration Energy Harvesters. Sensors.

[3] Alizzio D., Bonfanti M., Donato N., Faraci C., Grasso G.M., Lo Savio F., Montanini R., Quattrocchi A. (2021). Design and Performance Evaluation of a “Fixed-Point” Spar Buoy Equipped with a Piezoelectric Energy Harvesting Unit for Floating Near-Shore Applications. Sensors.

[4] Suresh K., Uma G., Santhosh Kumar B.V.M.P., Varun Kumar U., Umapathy M. (2011). Piezoelectric based resonant mass sensor using phase measurement. Measurement.

[5] Devoe D.L., Pisano A.P. (2001). Surface micromachined piezoelectric accelerometers. J. Microelectromech. Syst..

[6] Scheeper P., Gulløv J.O., Kofoed L.M. (1996). A piezoelectric triaxial accelerometer. J. Micromech. Microeng..

[7] Mohith S., Rao M., Karanth N., Kulkarni S.M., Upadhya A.R. (2021). Development and assessment of large stroke piezo-hydraulic actuator for micro positioning applications. Precis. Eng..

[8] Dabbagh V., Sarhan A.A.D., Akbari J., Mardi N.A. (2017). Design and manufacture of ultrasonic motor with in-plane and out-of plane bending vibration modes of rectangular plane with large contact area. Measurement.

[9] Gencoglu C., Özgüven H.N. (2014). Optimal Placement of Piezoelectric Patches on a Cylindrical Shell for Active Vibration Control BT. Top. Modal Anal..

[10] Yu X., Gao N., Xie Y., Zhang P., Long X. (2015). Displacement optimization of the path length control transducer for laser gyroscope by the finite element method. Int. J. Appl. Electromagn. Mech..

[11] Lee J. (2012). Mirror design for piezo-driven mechanical devices. Int. J. Precis. Eng. Manuf..

[12] Yu X., Long X. (2015). Parametric design of mechanical dither with bimorph piezoelectric actuator for ring laser gyroscope. Int. J. Appl. Electromagn. Mech..

[13] Das T.K., Shirinzadeh B., Al-Jodah A., Ghafarian M., Pinskier J. (2020). Computational parametric analysis and experimental investigations of a compact flexure-based microgripper. Precis. Eng..

[14] Wang F., Shi B., Huo Z., Tian Y., Zhang D. (2021). Control and dynamic releasing method of a piezoelectric actuated microgripper. Precis. Eng..

[15] Chow W.W., Gea-Banacloche J., Pedrotti L.M., Sanders V.E., Schleich W., Scully M.O. (1985). The ring laser gyro. Rev. Mod. Phys..

[16] Laser Gyroscope Leybold, LD Didactic Group. https://www.ld-didactic.de.

[17] Fenercioğlu T.O., Yalçinkaya T. (2019). Design optimization of a laser path length controller through numerical analysis and experimental validation. Int. J. Appl. Electromagn. Mech..

[18] Koper J.G., Ljungand B.H.G., Krupick J.G. (1994). Laser Length Control Assembly for Ring Laser Gyro. U.S. Patent.

[19] Zapotyl’Ko N.R., Katkovand A.A., Nedzvetskaya A.A. (2011). Piezo adjuster for compensating the thermal variations of the optical path length of the cavity of a laser gyroscope. J. Opt. Technol..

[20] MSC (2011). Software Corporation, Marc®2011 Volume A: Theory and User Information.

[21] (1988). IEEE Standard on Piezoelectricity, in ANSI/IEEE Std 176-1987.

[22] Piezodiscs, Material Specification Sheet, Fuji Ceramics. http://www.fujicera.co.jp.

[23] High-Resolution Digimatic Measuring Unit, Litematic VL-50-B/50S-B, Product Specification Sheet. https://www.mitutoyo.com.

[24] optoNCDT 2300 Laser Displacement Sensor Datasheet, Micro-Epsilon. https://www.micro-epsilon.com.

[25] Gençoğlu C. (2014). Active Vibration Control of Beams and Cylinrical Structures Using Piezoelectric Patches. Master’s Thesis.

